# The role of dexmedetomidine as an adjuvant for high-thoracic erector spinae plane block for analgesia in shoulder arthroscopy; a randomized controlled study

**DOI:** 10.1186/s12871-023-02014-2

**Published:** 2023-02-15

**Authors:** Mohamed Ahmed Hamed, Omar Sayed Fargaly, Rana Ahmed Abdelghaffar, Mohammed Ahmed Moussa, Mohammad Fouad Algyar

**Affiliations:** 1 Department of Anesthesiology, Faculty of Medicine, Fayoum University, Fayoum, 63511 Egypt; 2 Department of Orthopedics, Faculty of Medicine, Fayoum University, Faiyum, Egypt; 3 Department of Anesthesiology, Surgical Intensive Care and Pain Medicine, Faculty of Medicine, Kafrelsheikh University, Kafr el-Sheikh, Egypt

**Keywords:** Dexmedetomidine, Erector spinae plane block, Shoulder arthroscopy

## Abstract

**Background:**

Management of postoperative pain after shoulder arthroscopy is an important issue. Dexmedetomidine, as an adjuvant, improves nerve block efficacy and decreases postoperative consumption of opioids. As a result, we designed this study to determine if adding dexmedetomidine to an erector spinae plane block (ESPB) that is guided by ultrasound (US) is beneficial for treating immediate postoperative pain following shoulder arthroscopy.

**Methods:**

This randomized controlled double-blind trial recruited 60 cases 18–65 years old of both sexes, American Society of Anesthesiologists (ASA) physical status I-II, scheduled for elective shoulder arthroscopy. Random allocation of 60 cases was done equally into two groups according to the solution injected in US-guided ESPB at T2 before general anesthetic induction. Group (ESPB): 20 ml 0.25% bupivacaine. Group (ESPB + DEX): 19 ml bupivacaine 0.25% + 1 mL dexmedetomidine 0.5 µg/kg. The primary outcome was The total rescue morphine consumption in the first 24 postoperative hours.

**Results:**

The mean intraoperative fentanyl consumption was significantly lower in the group (ESPB + DEX) compared to the group (ESPB) (82.86 ± 13.57 versus 100.74 ± 35.07, respectively, *P* = 0.015). The median (IQR) time of the 1^st^ rescue analgesic request was significantly delayed in the group (ESPB + DEX) compared to group (ESPB) [18.5 (18.25–18.75) versus 12 (12–15.75), *P* = 0.044]. The number of cases that required morphine was significantly lower in the group (ESPB + DEX) than in the group (ESPB) (*P* = 0.012). The median (IQR) of total postoperative morphine consumption in 1^st^ 24 h was significantly lower in the group (ESPB + DEX) compared to the group (ESPB) [0 (0–0) versus 0 (0–3), *P* = 0.021].

**Conclusion:**

The dexmedetomidine as an adjuvant to bupivacaine in ESPB produced adequate analgesia by reducing the intraoperative and postoperative opioid requirements in shoulder arthroscopy.

**Trial Registration:**

This study is registered on ClinicalTrials.gov (NCT05165836; principal investigator: Mohammad Fouad Algyar; registration date: 21/12/ 2021).

## Introduction

Shoulder arthroscopy is one of the most frequently performed orthopedic surgery for various surgical applications, such as instability and stiffness of rotator cuff tears [1, 2]. Postoperative pain is an unfavorable outcome causing distress to cases. So, good pain management is critical to recovery after orthopedic surgery and is required to optimize surgical results [3].

There are several analgesic strategies and methods for postoperative pain management following a shoulder arthroscopy, including regional nerve blocks, intra-articular administration of local anesthetic (LA), non-steroidal anti-inflammatory drugs, intravenous narcotics shots, or patient-controlled analgesia (PCA), and continuous-flow cold therapy [4].

The erector spinae plane block (ESPB) is a relatively new regional anesthetic technique that can control acute pain for different surgeries [5]. Additionally, ESPB is a successful treatment for persistent pain in the shoulder, and the LA distribution was observed to extend to C3 if conducted at the T2 level [6–8].

Dexmedetomidine is an effective α2 agonist that lowers blood pressure, causes perioperative sympatholysis, and improves the anaesthesia produced by other anaesthetics [9–11]. Dexmedetomidine can also lengthen and speed up the onset of nerve blocks when used with LA [12–15].

We hypothesized that dexmedetomidine use as an adjuvant in ESPB in shoulder arthroscopy would provide more effective and prolonged pain relief.

Nevertheless, there is a paucity of studies that discussed the impact of dexmedetomidine use as an adjuvant in ESPB in shoulder arthroscopy, so we established this trial to evaluate the effect of adding dexmedetomidine to ultrasound (US)-guided ESPB for the management of acute postoperative pain in shoulder arthroscopy.

## Methods

This randomized controlled double-blind was conducted following the tenets of the Declaration of Helsinki. The study design was approved by the ethical review board of Kafrelsheikh University, Egypt (MKSU 40–11-21), and written informed consent was reported from all cases. The study was conducted after registration on ClinicalTrials.gov (NCT05165836; principal investigator: Mohammad Fouad Algyar; date of registration: December 21, 2021, with no plan to share individual participant data). Our research enrolled 60 cases 18 to 65 years old, of both sexes, with body mass index < 40 kg/m^2^, American Society of Anesthesiologists (ASA) physical status I-II, scheduled for elective shoulder arthroscopy. The study was conducted from December 21, 2021, to May 20, 2022, at Kafrelsheikh and Fayoum University hospitals.

Exclusion criteria were allergic history to drugs used in the trial, shoulder diagnostic arthroscopic operations, chronic opioid usage, skin infection of the block area, and coagulopathy.

### Randomization and blindness

Sixty cases were randomly divided into two groups by a computer-generated sequence into sealed opaque envelopes. Group (ESPB) *n* = 30: received ESPB by 20 ml 0.25% bupivacaine, group (ESPB + DEX) *n* = 30: received ESPB by 19 ml bupivacaine 0.25% + 1 ml dexmedetomidine 0.5 μg/kg. Cases, anesthesiologists, and outcome assessors were blinded. A devoted pharmacist formulated the research solutions without further involvement in the trial. Unaware of the group assignment, a second anesthesiologist examined intraoperative and postoperative parameters.

Preanesthetic assessment included history taking, general examination, and laboratory investigations.

All cases were premedicated by midazolam 2 mg intravenous (IV) after cannula insertion. In addition, cases underwent US-guided ESPB before general anesthesia induction (GA).

### High-Thoracic ESPB Technique [7]

Blocks were put together using a US machine (Philips CX50 Extreme edition). To see the lateral tip of the T2 transversal process, a 2–5 MH2 curved probe was positioned transversely. The patient's transducer was positioned longitudinally three cm laterally of the T2 spinous process. The hyperechoic transverse process cast a shadow across the muscles of the rhomboid major, trapezius, and erector spinae. The skin was thereafter numbed with 3 ml of 2% lidocaine. A 20-gauge needle was used to administer 20 ml of 0.25% bupivacaine or 19 ml of bupivacaine 0.25% + 1 mL of 0.5 g/kg dexmedetomidine to the designated group. The tip of the needle was positioned in the deep fascial plane (anterior) side of the erector spinae. The fluid spreading out, lifting the erector spinae muscle away from the transverse process shadow, revealed the needle's position.

The success of the block was confirmed by the loss of pinprick sensation on the dermatomal site of the block after 30 min of injection. Therefore, cases of failed blocks were excluded from the study.

The conventional approach of GA was used in every case. Monitoring was done by pulse oximetry, noninvasive blood pressure, temperature probe, capnography, and 5- lead ECG. Propofol 2 mg/kg IV and fentanyl one µg/kg IV were used to induce GA. Cis-atracurium 0.15 mg/kg IV was used to facilitate endotracheal intubation. Isoflurane 1–1.5% in 50% oxygen was used for anesthesia maintenance. Increasing dosages of 0.03 mg/kg cis-atracurium were administered intravenously. The depth of anesthesia was adjusted to obtain an adequate level of anesthesia by titrating the concentration according to the BIS monitoring (BIS Complete Monitoring System) to keep the BIS value between 40 and 60. Supplemental dosages of fentanyl one µg/kg IV were administered whenever the mean arterial blood pressure (MAP) or heart rate (HR) was over 20% from baseline readings. Intraoperative fentanyl (including induction dose) and isoflurane consumption were recorded. Intraoperative HR and MAP were measured intraoperatively every 15 min.

The extubation was completed once the anaesthesia was stopped. The post-anesthesia care unit received the cases (PACU). After then, cases were moved to the ward. Cases received a 1 gm/8 h IV dose of paracetamol. If the numeric rating scale (NRS) score is ≥ 4, rescue analgesia in the form of 3 mg IV morphine was administered. This procedure was repeated with a 10-min lockout interval until the NRS value dropped below 3. It was noted when the first rescue analgesia was used and how much morphine was used overall in the first day after surgery. In the PACU, immediately after surgery, and at 1, 2, 4, 6, 8, 12, and 24 h postoperatively, pain was assessed using the numerical rating scale (NRS), HR, and MAP.

Adverse events were recorded, such as nausea, vomiting, and hypotension (MAP < 20% of baseline readings and was managed by ephedrine, bradycardia (HR < 60 beats/min and was managed by atropine).

### Outcomes

The total postoperative morphine consumption was our primary outcome. Secondary outcomes were postoperative pain scores, time to first request for rescue analgesia, and any adverse events.

### Sample size calculation

G*Power 3.1.9.2 (Universitat Kiel, Germany) was used to determine the sample size. The mean (± SD) total morphine consumption (our primary outcome) was 3.43 ± 2.56 mg in ESPB without dexmedetomidine and 1.71 ± 1.79 mg in ESPB with dexmedetomidine according to a previous study (15). In each group, 30 cases were recruited with 0.779 effect size, 95% confidence limit, and 80% power, group ratio 1:1, and three cases were added to overcome dropout.

### Statistical analysis

SPSS v26 was used to perform statistical analysis (IBM©, Chicago, IL, USA). Using the Shapiro-Wilks test and histograms, the normality of the data distribution was determined. Parametric quantitative data were expressed as mean and standard deviation (SD) and analyzed by unpaired student t-test. Non-parametric quantitative data were expressed as the median and interquartile range (IQR) and analyzed using the Mann–Whitney test. Qualitative variables were given as frequency and percentage (%) and analyzed using the Chi-square or Fisher's exact test. A two-tailed *P* value less than or equal to 0.05 was deemed statistically significant.

## Results

**Fig. 1:**
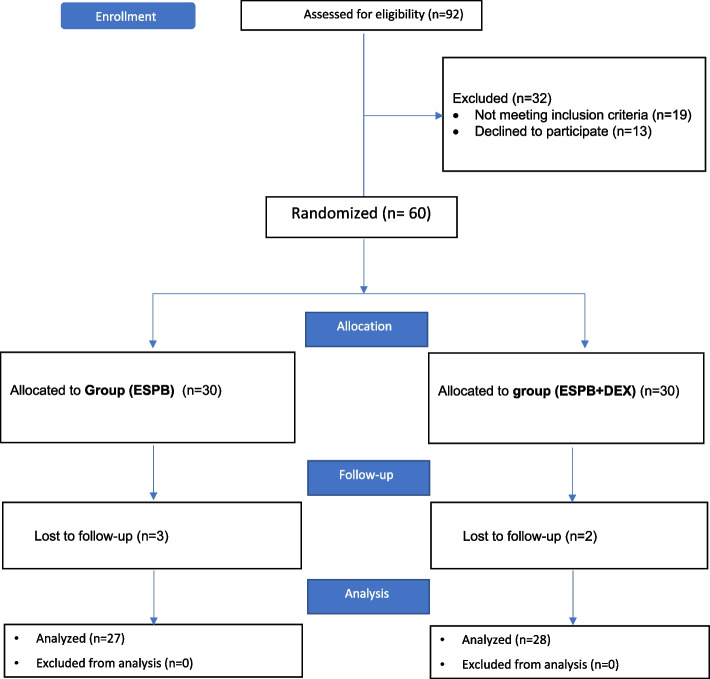
Consort flow diagram of the study population

In this trial, eligibility was determined for 92 cases. Sixty cases were divided into two groups of equal size. Three cases in the group (ESPB) and two cases in the group (ESPB + DEX) were dropped out due to failed block. Only 55 cases were analyzed (Fig. 1).

Insignificant differences were found between the two groups regarding patient demographics and duration of surgery (Table 1).

**Table 1:** Patient characteristics and duration of surgery of the studied groups

		Group ESPB	Group ESPB + DEX	*P* value
**Sample size, n**		30	30	
**Mean Age (SD) in (years)**		44.81 ± 13.62	41.11 ± 12.98	0.306
**Mean BMI (SD) in (kg/m**^**2**^)		32.42 ± 4.9	31.79 ± 6.11	0.675
**Gender, n (%)**	**Male**	23 (85.2%)	22 (78.6%)	0.746
	**Female**	4 (14.8%)	6 (21.4%)	
**ASA physical status, n (%)**	**I**	21 (77.78%)	20 (71.43%)	0.770
	**II**	6 (22.22%)	8 (28.57%)	
**Mean duration of surgery (SD) in (min)**		119.7 ± 28.09	125 ± 35.47	0.543

Data presented as mean ± SD and frequency (%), *BMI* Body mass index, *ASA* American Society of Anesthesiologists

The mean intraoperative fentanyl consumption was significantly lower in the group (ESPB + DEX) compared to the group (ESPB) (82.86 ± 13.57 versus 100.74 ± 35.07, respectively, *P* = 0.015). The median (IQR) time of the 1^st^ rescue analgesic request was significantly delayed in the group (ESPB + DEX) compared to the group (ESPB) [18.5 (18.25–18.75) versus 12 (12–15.75), *P* = 0.044]. The number of cases that required morphine was significantly lower in the group (ESPB + DEX) than in the group (ESPB) (*P* = 0.012). The median (IQR) of total postoperative morphine consumption in 1^st^ 24 h was significantly lower in the group (ESPB + DEX) compared to the group (ESPB) [0 (0–0) versus 0 (0–3), *P* = 0.021] (Table 2).

**Table 2:** Intraoperative fentanyl, isoflurane consumption, and postoperative total morphine consumption in both groups

	**Group** ESPB	**Group** ESPB + DEX	***P* value**
**Sample size,n**	27	28	
**The mean intraoperative fentanyl consumption(SD) in (µg)**	100.74 ± 35.07	82.86 ± 13.57	0.015
**Time to the 1**^**st**^** rescue analgesic request (IQR) in hours**	12 (12–15.75)	18.5 (18.25–18.75)	0.044
**The cases required postoperative morphine (SD)**	11(40.74%%)	2 (7.14%%)	0.012
	(*n* = 11)	(*n* = 2)	
**The Postoperative total morphine consumption in 1**^**st**^** 24 h (IQR) in (mg)**	0 (0–3)	0 (0–0)	0.021

Data presented as mean ± SD or median (IQR), or frequency (%)

NRS was significantly lower in the group (ESPB + DEX) compared to the group (ESPB) at 12 and 18 h (*P* value = 0.001 and 0.001 respectively) and insignificantly different between both groups at other measurement times (Table 3).

**Table 3:** Comparison of the Numerical Rating Scale of the studied groups

	**Group** ESPB	**Group** ESPB + DEX	***P* value**
**Sample size, n**	27	28	
**Immediate in PACU**	1 (1–2)	1 (0–1)	0.098
**1 h**	1 (1–2)	1 (0–2)	0.415
**2 h**	1 (1–2)	2 (0–3)	0.192
**4 h**	2 (1–3)	2 (0–3)	0.479
**6 h**	1 (0–2)	2 (0.75–3)	0.276
**8 h**	1 (0–2)	2 (0.75–2.25)	0.289
**12 h**	3 (2.5–4)	1 (0–2)	< 0.001
**18 h**	3 (2–3)	2 (1–2.25)	< 0.001
**24 h**	1 (0–1.5)	1 (0–2)	0.719

Data presented as median (IQR)

Intraoperative HR and MAP were insignificantly different between both groups in all measurements. However, HR and MAP postoperatively were significantly lower in the group (ESPB + DEX) than in the group (ESPB) at 12 and 18 h (*P* value < 0.05) and compared between groups at other measurements.

PONV, bradycardia, and hypotension were insignificantly different between both groups. Block-related complications did not occur in any patient (Table 4).

**Table 4:** Adverse effects of the studied groups

	**Group** ESPB	**Group** ESPB + DEX	***P* value**
**Sample size,n**	27	28	
**PONV, n (%)**	6 (22.2%)	4 (14.3%)	0.730
**Bradycardia, n (%)**	3 (10%)	5 (17%)	0.706
**Hypotension, n (%)**	2 (7%)	4 (13%)	0.670

Data presented as frequency (%), *PONV* Postoperative nausea and vomiting

## Discussion

In the current research, we observed that adding dexmedetomidine in US-guided ESPB was associated with a better analgesic effect by reducing intraoperative fentanyl and postoperative morphine consumption and a more prolonged analgesic effect and reduction of NRS with stable hemodynamics.

Peripheral nerve blocks, such as interscalene and supraclavicular blocks, can be utilised for postoperative shoulder analgesia. There are many methods for treating pain following shoulder surgery [16]. ESPB is an alternative method for the management of shoulder postoperative pain [7], and it has also been reported that ESPB may be used for chronic shoulder pain and upper extremity surgery [6, 8].

The increase in cation channels brought on by hyperpolarization, which prevents the nerve's membrane potential from returning to its resting state for potential discharge after hyperpolarization, is what gives perineuronal dexmedetomidine its analgesic effects [17]. Compared to placebo, perineural dexmedetomidine showed 60% prolongation of ulnar nerve sensory blockage, while systemic dexmedetomidine prolonged the duration of sensory block by 10% [18].

This is similar to another randomized controlled trial done by Elshal et al., [19] who documented that the addition of dexmedetomidine 0.5 µg/kg to bupivacaine in US-guided ESPB enhanced analgesia in open thoracotomy; this was evidenced by extended analgesic duration and a lower Visual Analog Scale at rest and while coughing and both intraoperative fentanyl and postoperative morphine use in 24 h with comparable hemodynamic characteristics in both groups. Dexmedetomidine's analgesic effects are regulated by various processes [20].

Similarly, Ciftci et al. [7] stated that performing high thoracic, high-volume, single-injection ESPB may provide effective analgesia as part of a multimodal analgesia treatment following arthroscopic shoulder surgery.

In contrast Kapukaya et al. [8] in their study Interscalene brachial plexus block offers more effective pain control than ESPB after arthroscopic shoulder surgery, according to a study comparing erector spinae plane block with interscalene brachial plexus block for postoperative analgesic management in patients who underwent shoulder arthroscopy.

Shanthanna et al. [21] in their randomised controlled trial; After arthroscopic shoulder surgery, the erector spinae plane block is preferred to peri-articular injection for pain management. They came to the conclusion that ESPB conducted at T2 was not superior to peri-articular injection for pain control and narcotic intake in major arthroscopic shoulder surgery. Also, previous research [22] showed that adding dexmedetomidine 0.5 g/kg to ropivacaine 0.25% for transversus abdominis plane block lowered the opioid intake and prolonged the analgesia duration without causing any extra adverse events following a variety of major surgeries.

Also, Mohta et al. [23] found that adding dexmedetomidine one µg/kg in paravertebral block (PVB) to bupivacaine 0.5% showed decreased morphine consumption and pain scores and delayed time to request the first analgesia in cases undergoing extensive surgery for breast cancer.

In addition, Mohamed et al. [24] found that in cases taking dexmedetomidine1 µg/kg, the time required first to rescue analgesia was longer, and the mean total intake of opioids was lower utilizing thoracic PVB with 20 ml of 0.25% bupivacaine in modified radical mastectomy.

According to our trial's results, adding dexmedetomidine 0.5 µg/kg to ESPB did not generate significant changes in HR or MAP. In addition, the incidence of other adverse events, such as nausea, vomiting, hypotension, and bradycardia, was comparable across the two groups.

Nevertheless, Esmaoglu et al. [25] documented that adding dexmedetomidine 100 µg to the LA resulted in significant HR, systolic blood pressure, and diastolic blood pressure within the first two hours. Other studies [26, 27] have reported marked bradycardia and hypotension following dexmedetomidine administration. Our research did not observe that we used a lower dose of dexmedetomidine (0.5 µg/kg).

### The limitations

There were limitations in our trial, including a relatively small sample size to prove the occurrence of adverse effects of the block and a limited follow-up period (only 24 h). the sample size calculation was based on the previous study conducted on thoracotomies, in which many other factors are associated with postoperative pain. We used a volume of 20 ml 0.25% bupivacaine. More studies may be performed with different volumes and concentrations. Also, no control group utilized systemic analgesia or one of the regional gold-standard approaches in shoulder arthroscopy. More extensive randomized trials are needed to determine the dexmedetomidine effect on chronic pain and other related complications.

## Conclusion

The dexmedetomidine as an adjuvant to bupivacaine in ESPB produced adequate analgesia by reducing the intraoperative and postoperative opioid requirements in shoulder arthroscopy.

## Data Availability

The datasets used and analyzed during the current study are available from the corresponding author upon reasonable request.

## Abbreviations

ASA: The American Society of Anesthesiologists
BIS: Bispectral Index
BMI: Body mass index
CI: Confidence Interval
ESPB: Erector Spinae Plane Block
GA: General Anesthesia
HR: Heart Rate
IQR: Interquartile Range
LA: Local anesthetic
MAP: Mean Arterial Blood Pressure
NRS: Numeric Rating Scale
PACU: Post Anesthesia Care Unit
PCA: Patient-Controlled Analgesia
PONV: Postoperative nausea and vomiting
PVB: Paravertebral Block
SD: Standard deviation
US: Ultrasound
